# Changes in the fatty acid profile of fish oil derived from Pangasius catfish *(Pangasianodon hypophthalmus) *processing waste due to variations in fish size and heating temperatures

**DOI:** 10.12688/f1000research.141714.1

**Published:** 2023-10-02

**Authors:** Netti Aryani, Indra Suharman, Benny Heltonika, Edison Edison, Andarini Diharmi

**Affiliations:** 1 Department Aquaculture, Faculty of Fisheries and Marine, Universitas Riau, Pekanbaru, Riau, 28293, Indonesia; 2 Department of Fishery Products Processing Faculty of Fisheries and Marine, Universitas Riau, Pekanbaru, Riau, 28293, Indonesia

**Keywords:** Pangasius catfish, Biometry group, Abdominal fat, Heating temperatures, Fatty acid

## Abstract

Abstract

**Background**: During the last decade, the demand for fish oil as a feed component has increased. Therefore, identifying sources of fish oil from processed catfish waste is an important task. This study aimed to analyse the relationship between fresh weight and mesenteric weight in each group of fish and determine how variations in the size of catfish ( *Pangasianodon hypophtalmus*) and heating temperature affect fatty acid profiles.

**Methods:** The primary source of raw material used to produce fish oil is the mesenteric organ, specifically the belly fat of catfish. This material was obtained from catfish in the following categories: Group A (290-390 g), Group B (440-685 g), and Group C (890-1,100 g). The fish oil was subjected to four different levels of heating temperature (45 ^o^C, 60 ^o^C, 75 ^o^C, and 90°C). The parameters that were analysed included biometry measurements, the correlation between fish weight and mesenteric tissue, and fatty acid content.

**Results:** Significant positive linear correlations were found between body weight and mesenteric tissue in Group A ( *p* < 0.001, *r* ^2^ = 0.65), Group B ( *p* < 0.001, *r* ^2^ = 0.72), and Group C ( *p* < 0.001, *r* ^2^ = 0.64). Notably, significant differences in fatty acid composition were observed among fish groups and varied heating temperatures. Within the fish group, unsaturated fatty acids ranged from 51.25% to 56.61%, n-3 fatty acids from 1.44% to 1.77%, n-6 fatty acids from 9.04% to 10.1%, and n-9 fatty acids from 35.35% to 37.43%. Temperature fluctuations led to unsaturated fatty acid contents of 52.06% to 55.55%, n-3 fatty acids of 1.28% to 1.46%, n-6 fatty acids of 8.14% to 8,45%, and n-9 fatty acids of 34.9% to 36.92%.

**Conclusions:** The best fatty acid composition in fish oil was found in Group B (with a weight between 440 g to 685 g) through a heating process at 45°C

## Introduction

The global rise in population and improvements in quality of life have transformed global dietary patterns, leading to a transition towards animal-based products, such as farmed finfish and crustaceans. ^1^^,^^2^ A key approach to fostering sustainability in aquaculture involves reducing the reliance on ingredients derived from marine natural resources, such as fish meal and fish oil. ^3^^–^^5^

In 2020, the global utilization of fish oil in fish feed is projected to reach approximately one million tons. ^6^ Furthermore, the demand for fish oil as fish feed is expected to exhibit a compound annual growth rate (CAGR) of approximately 5.2% from 2021 to 2028. ^7^ Hence, scientists have assessed the impacts of substituting fish oil with a combination of *Schizochytrium* sp. and *Microchloropsis gaditana*, including camelina oil, on the growth and efficiency of fish feed utilization. ^8^^–^^10^ They have also explored the utilization of high-DHA algae meals as a viable substitute for high-DHA fish oil. ^11^^,^^12^

Oil derived from the adipose tissue of freshwater fish is utilized as a dietary supplement to enhance the reproductive capabilities of broodfish and improve their reproductive performance. ^13^ Furthermore, beef tallow oil has been incorporated into the diets of juvenile turbot, acting as a substitute for fish oil and providing an alternate source of nutrition. ^14^ In addition, sesame oil meal, a byproduct rich in nutritional value from the food industry, is applied to generate sustainable aquaculture feeds. ^15^ For example, linseed oil is used as a complete replacement for fish oil in formulated diets. ^16^

Our hypothesis postulates that that differences in group fish size and heating temperatures influence the fatty acid composition of mesenteric tissue within Pangasius catfish processing remnants. The objectives of this research were as follows: (i) to analyse the relationship between fresh body weight and mesenteric tissue weight in each sample group of fish, (ii) to analyse how variations in Pangasius catfish size groups impact the fatty acid content in fish oil generated from mesenteric tissue within pangasius catfish processing waste, and (iii) to investigate the influence of fluctuating heating temperatures on the fatty acid content within the oil produced during the processing of Pangasius catfish mesenteric tissue.

## Methods

### Ethical statement

This research was carried out within the framework of a project entitled ‘Changes in the Fatty Acid Profile of Fish Oil Derived from the Processing Waste of Pangasius Catfish ( *Pangasianodon hypophthalmus*) Due to Variations in Fish Size and Heating Temperature’.

The Research Ethics and Community Service Board of the University of Riau has approved the collection of Pangasius catfish and catfish processing waste by following the ARRIVE guidelines, as stated in grant letter No. 2439/UN19.5.1.3/AL.04/2023, dated 10 June 2023. It is important to note that the Pangasius catfish is not included in the protected species category according to Indonesian laws and regulations. In addition, Pangasius catfish is a species cultivated en masse in Indonesia to be processed into ready-to-eat food in restaurants and as smoked fish. Efforts to treat this species produce waste that is recommended to be utilized with a green economy approach to prevent adverse environmental impacts.

All of this research was conducted at the Processing Laboratory, Faculty of Fisheries and Maritime Affairs, University of Riau, and the reporting was in accordance with ARRIVE's guidelines. It commenced in June and is scheduled for completion in July 2023.

### Physical characteristics of fish samples

Sixteen live Pangasius catfish ( *Pangasianodon hypophthalmus*) from each size group were sourced from freshwater earthen ponds in Koto Mesjid Village, in Kampar Regency, Riau Province, Indonesia. The selection of the sample size was guided by the typical group of fish processed by local smoked fish producers. Fresh fish were carefully stored on ice and immediately transported to the laboratory. The Pangasius catfish samples lived in each size group before their mesenteric parts are collected. The fish samples were euthanized by puncturing their brain with a 20G x 1” syringe needle.

Each fish was weighed individually (TW, g) and their standard length (SL, cm) and maximum height (H, cm) were measured. The measurement of standard length involved assessing from the mouth to the termination point of the upper part of the caudal fin, while height was determined in a vertical manner, excluding the fins. Furthermore, the condition factor (CF) was computed by applying the formula CF = (TW/SL ^3^) x 100.

### Preparation of fish oil raw material

The primary source of raw material for fish oil production is the mesenteric organ, specifically the belly fat of catfish. This material is sourced from the following distinct size categories: Group A (weighing between 290 g to 390 g), Group B (weighing between 440 g to 685 g), and Group C (weighing between 890 g to 1,100 g). These catfish are procured from a local community engaged in processing Pangasius catfish. The intention behind this collection is to eventually process the fish into smoked products within Koto Mesjid Village, which is located in the Kampar Regency of Riau Province, Indonesia.

**Figure 1.**
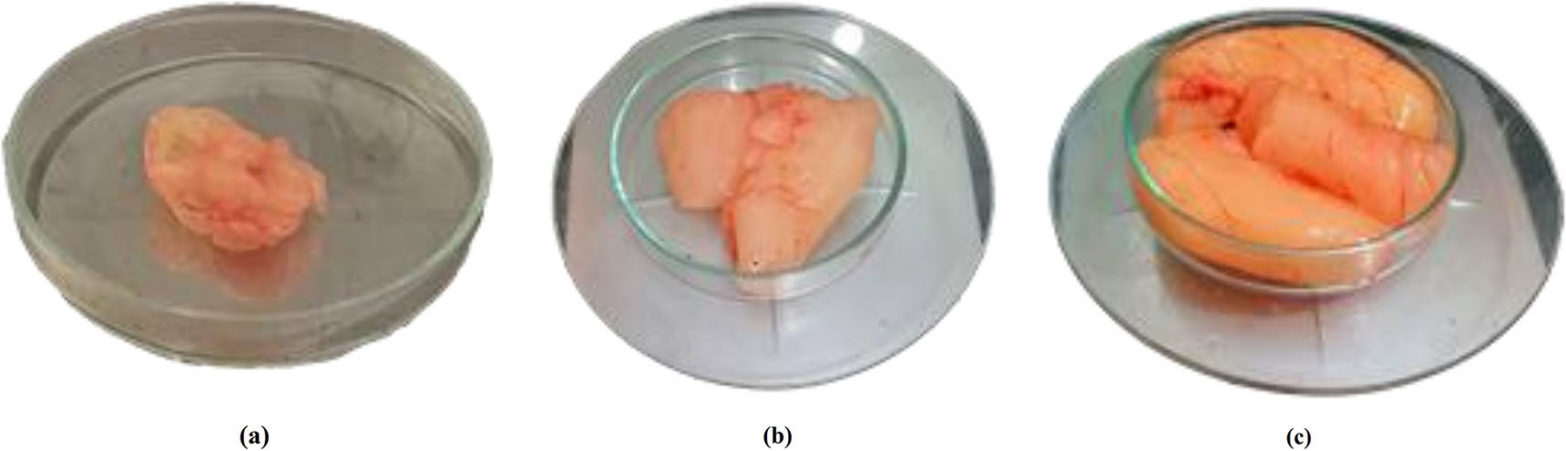
The mesenteric tissue of Pangasius catfish, where (a) represents group A; (b) represents group B, and (c) represents group C.

Every individual fish underwent a procedure wherein its belly fat (mesenteric section) was extracted using a knife. Subsequently, the collected fat was weighed using an analytical balance model, specifically the OHAUS CT 6000i. Following this step, the mesenteric fat was carefully cleansed using a tissue to eliminate any dirt or blood residue. The fat obtained from catfish of different sizes was segregated and placed within a cooling container filled with ice cubes. This fat was then transported to the Fish Hatchery and Breeding Laboratory, which is housed within the Faculty of Fisheries and Marine Affairs at the University of Riau. The mesenteric tissue morphometry of each group of fish is presented in Figure 1.

After the samples were delivered to the laboratory, the size of the belly fat was reduced (ranging from 1-1.5 cm) using a knife. Belly fat was extracted by heating it in an electric oven (model: VIENTA Smart Oven OVN.V-CZ30E, Mode in China) at 60°C for one hour. The results of the extraction in the form of liquid oil from various fish size groups were filtered using a fine oil filter, stainless, with a θ13 cm. All the oil produced was placed into a closed container and then analysed for crude fat content and fatty acid profile at the Testing and Calibration Services Laboratory, IPB University.

### Varied temperatures applied to the fish oil

The purpose of this experiment is to determine how temperature variations can affect the properties and characteristics of fish oil. This experiment was carried out by heating fish oil at the following different temperatures: 45°C, 60°C, 75°C, and 90°C.

First, the fish oil was heated using an electric heater equipped with accurate temperature control. When it reached 45°C, the fish oil was heated to that point and maintained at that temperature for 60 minutes. The same process was repeated at 60°C, 75°C and 90°C. Each heating step was carefully monitored to ensure consistent and precise temperatures.

After the heating process was complete, the fish oil at each temperature was observed to observe chemical changes in the fatty acid composition due to temperature variations. The results from these experiments will provide valuable insight into how temperature can affect the quality and stability of fish oil.

Furthermore, the data obtained from this experiment will be analysed comprehensively to understand its practical implications. The information obtained can be used to optimize the process of heating fish oil in various applications, including in fish feed enrichment. As such, this experiment shows potential to improve efficiency and quality in the fishing and feed industries.

### Analysis of fatty acids

The fatty acid composition of the fish oil from each treatment was assessed utilizing the gas chromatography–mass spectrometry (GC–MS) technique. Extraction of total lipids was executed in accordance with the procedure adopted from Folch *et al*. (1957), as detailed by Rajion, ^17^ employing a solvent system composed of chloroform and methanol in a ratio of 2.1 (v/v). Transmethylation, a process involving 14% methanolic boron trifluoride, was subsequently carried out.

### Statistical analysis

The Statistical Package for the Social Science ( SPSS) 16.0 software package (SPSS; Chicago, IL) was employed for the data analysis process. To assess data homogeneity, Levin's test was conducted. One-way ANOVA was utilized to examine the impact of treatments, followed by the post hoc Duncan's multiple range test. ^18^ The presented data are expressed as the mean value ± standard error. Regression curve estimation was utilized to generate the Figures.

## Results and discussion

### Biometry measurement

This study presents the mean fresh weight, standard length, body height, condition factor, and mesenteric tissue weight for each fish group, as outlined in Table 1. ^54^ Notably, statistically significant differences ( *p*<0.05; see Table 1) were observed in the wet weight, standard length, height, condition factor, and mesenteric tissue weight among the examined fish groups.

**Table 1.** Biometry of Pangasius catfish, *Pangasianodon hypophthalmus.*

Biometry	Group A	Group B	Group C
Weight (g)	343.00 ± 8.38 ^a^	577.33 ± 29.79 ^b^	976.73 ± 26.60 ^c^
Standard length (cm)	27.82 ± 0.14 ^a^	30.46 ± 1.88 ^b^	36.69 ± 0.54 ^c^
Height (cm)	6.14 ± 0.09 ^a^	7.44 ± 0.12 ^b^	9.68 ± 0.12 ^c^
Condition factor	1.58 ± 0.02 ^a^	2.1 ± 0.28 ^b^	1.98 ± 0.04 ^c^
Mesenteric weight (g)	5.8 ± 0.03 ^a^	14.00 ± 2.37 ^b^	45.81 ± 8.19 ^c^

Note: Group A: 290 g to 390 g; Group B: 440 g to 685 g; Group C: 890 g to 1100 g.

Mean ± SE (n = 3) with different letters in the same row are significantly different.

The notion of habitat stability, which contributes to species' overall well-being and resilience, can be comprehended by assessing the condition factor. The condition factor (K), derived from the interplay between length and weight, is frequently employed to gauge the health status of fish. ^19^ Fishes displaying a lower condition index are commonly presumed to have encountered unfavourable environmental circumstances or insufficient nourishment. ^20^

In the current study, the condition factor of collected animal samples ranged from 1.58 to 2.1. This range indicates that the Pangasius catfish are maintained within an optimal environment and are provided with adequate nourishment during their rearing. A condition factor equal to or exceeding one underscores a favourable scenario, denoting satisfactory feeding levels and appropriate environmental conditions. ^21^

### The correlation between body weight and mesenteric weight

**Figure 2.**
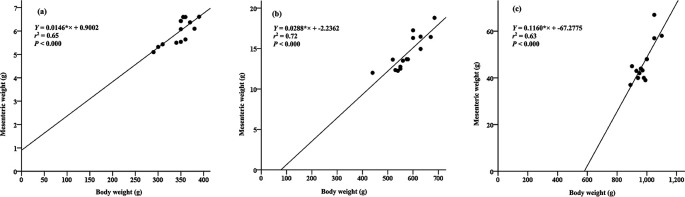
The relationships between body weight and mesenteric weight for different groups of Pangasius catfish under cultivation: Group A (a), Group B (b), and Group C (c).

A linear relationship in each weight group exhibits the correlation between body weight and mesenteric weight in Pangasius catfish. Specifically, for Group A, the relationship is represented as y = 0.0146*× + 0.9002 (with an *r* ^2^ value of 0.65, as seen in Figure 2(a)). In Group B, the relationship is expressed as y = 0.0288*× + -2.2362 (with an *r* ^2^ value of 0.72, depicted in Figure 2(b)). Last, for Group C, the relationship is characterized by y = 0.1160*× + -67.2775 (with an *r* ^2^ value of 0.64, displayed in Figure 2(c)). Notably, group A and C fish moderately correlate with mesenteric tissue weight, while group B fish strongly correlated with mesenteric tissue weight.

The waste generated from the Pangasius catfish processing home industry can account for up to 76% of the total weight of the processed fish. This waste comprises various parts, including the head, skin, entrails, tail, bones, and belly flap. ^22^^,^^23^ On the other hand, the processing of smoked Pangasius catfish results in approximately 7.67% waste. This waste category encompasses stomach contents, abdominal fat, and internal digestive organs. Moreover, abdominal fat contributes approximately 20.07% of the weight of the stomach contents. ^24^ This study revealed that the average mesenteric tissue fat weight in each fish was as follows: Group A (1.68 ± 0.14%), Group B (2.42 ± 0.28%), and Group C (4.67 ± 0.62%). This observation demonstrates that as the sampled fish's weight increases, the mesenteric tissue's weight also increases.

### Fatty acid profiles of Pangasius catfish groups

The composition of fatty acids in the fish oil extracted from the mesenteric tissue of the three Pangasius catfish body weight groups is presented in Table 3. Differences in the fatty acid composition were noticeable among the three fish group sizes, with statistically significant variances ( *p* < 0.05) observed among the groups.

Palmitic acid (C16:0) was found to be present at higher levels in fish oil from fish group C, compared to those from groups A and B. Substantial evidence indicates that the elevated content of palmitic acid might have contributed to the decreased levels of eicosapentaenoic acid (EPA) in group C fish. Palmitic acid, in conjunction with other saturated fats (SFA), is recognized for its propensity to raise cholesterol levels in the serum of Nile tilapia. ^25^ EPA, at the very least, has the potential to mitigate some of the adverse impacts induced by palmitic acid. ^26^

In the different size categories of fish, significant quantities of mono-unsaturated fatty acids (MUFAs) were present, particularly oleic acid. The respective proportions of oleic acid were 32.72% for Group A, 36.61% for Group B, and 34.55% for Group C. Additionally, elaidic acid concentrations ranged from 13.72% to 16.31%, surpassing other classified MUFA types (refer to Table 2). This discovery aligns with previous data from *Sardinella lemuru* and *Cyprinus carpio* fish oil research, ^24^^,^^27^ in which oil from both species exhibited comparatively elevated levels of oleic and elaidic acid. Unlike the results found in research by Sattang et al., ^13^ fish oil derived from the adipose tissue of a freshwater hybrid catfish ( *P. gigas* x *P. hypophthalmus*) has an oleic acid content of 42.27%, while the elaidic acid content is low, namely, 0.25%.

**Table 2.** Fatty acid composition of Pangasius catfish oil with different body weights.

Fatty acid (% ww/ww)	Fish groups		
	A	B	C
Undecanoic acid (C11:0)	1.16 ± 0.02 ^a^	1.96 ± 0.02 ^b^	1.53 ± 0.10 ^c^
Tridecanoic acid (C13:0)	2.76 ± 0.01 ^a^	2.93 ± 0.00 ^b^	3.10 ± 0.01 ^c^
Palmitic acid (C16:0)	2.93 ± 0.01 ^a^	2.34 ± 0.02 ^b^	3.86 ± 0.00 ^c^
Behenic acid (C22:0)	0.97 ± 0.01 ^a^	1.53 ± 0.01 ^b^	1.29 ± 0.08 ^c^
Heneicosanoic acid (C21:0)	0.48 ± 0.02 ^a^	0.61 ± 0.03 ^b^	0.63 ± 0.15 ^c^
**Saturated fatty acids (SFAs)**	**8.32 ± 0.03**	**9.37 ± 0.06**	**10.42 ± 0.21**
Myristoleic acid (C14:1)	0.16 ± 0.01 ^a^	0.23 ± 0.02 ^b^	0.18 ± 0.08 ^c^
Pentadecanoic acid (C15:1)	8.31 ± 0.02 ^a^	8.13 ± 0.02 ^b^	7.84 ± 0.05 ^c^
Palmitoleic acid (C16:1)	0.23 ± 0.01 ^a^	0.17 ± 0.01 ^b^	0.15 ± 0.01 ^c^
Heptadecanoic acid (C17:1)	3.61 ± 0.01 ^a^	3.58 ± 0.01 ^b^	2.95 ± 0.05 ^c^
cis-9-Oleic acid (C18:1n9c)	32.72 ± 0.03	36.61 ± 0.20	34.55 ± 0.07
Eicosenoic acid (C20:1)	0.93 ± 0.01 ^a^	0.58 ± 0.04 ^b^	0.50 ± 0.05 ^c^
Nervonic acid (C24:1)	0.36 ± 0.01 ^a^	0.24 ± 0.00 ^b^	0.30 ± 0.01 ^c^
Elaidic acid (C18:1n9t)	13.72 ± 0.34 ^a^	16.31 ± 0.14 ^b^	13.89 ± 0.02 ^c^
**Mono-unsaturated fatty acids (MUFAs)**	**59.86 ± 0.63**	**65.97 ± 0.64**	**60.23 ± 0.15**
Linolelaidic acid (C18:2n9t)	9.34 ± 0.16 ^a^	9.52 ± 0.02 ^b^	8.53 ± 0.03 ^c^
Linoleic acid (C18:2n6c)	0.08 ± 0.00	0.06 ± 0.00	0.08 ± 0.00
Arachidonic acid (C20:4-n6)	0.31 ± 0.01 ^a^	0.52 ± 0.02 ^b^	0.43 ± 0.01 ^c^
Linolenic Acid, C18:3n6	0.10 ± 0.00 ^a^	1.22 ± 0.02 ^b^	0.84 ± 0.01 ^c^
Eicosatrienoic Acid, C20:3n3	0.18 ± 0.1 ^a^	0.24 ± 0.02 ^b^	0.35 ± 0.02 ^c^
Eicosapentaenoic acid (20:5n3)	0.10 ± 0.01 ^a^	0.15 ± 0.01 ^b^	0.12 ± 0.02 ^c^
Docosahexaenoic acid (C22:6n3)	0.11 ± 0.02 ^a^	0.16 ± 0.00 ^b^	0.13 ± 0.01 ^c^
**Poly-unsaturated fatty acids (PUFAs)**	**10.24 ± 0.31**	**11.88 ± 0.08**	**10.50 ± 0.11**
Unsaturated fatty acid	51.25	56.61	52.87
n-3 fatty acid	0.49	1.77	1.44
n-6 fatty acid	9.73	10.1	9.04
n-9 fatty acid	33.56	37.43	35.35

Note: Group A: 290 g to 390 g; Group B: 440 g to 685 g; Group C: 890 g to 1100 g.

Mean ± SE (n = 3) with different letters in the same row are significantly different.

Elaidic acid is an isomer of oleic acid, which has a trans configuration. ^28^ Based on the 2021 regulations of the Food and Drug Monitoring Agency of the Republic of Indonesia, the amount of trans fatty acids in oil cannot exceed 2%. ^29^ Similarly, European Food Safety Authority, ^30^ elaidic acid in food cannot exceed 2%. Therefore, fish oil rich in elaidic acid must undergo a purification step to reduce the levels of trans fatty acids before it can be used. Trans fatty acids in fish oil arise due to high-temperature heating when canning fish, changing the fatty acid structure from the natural cis form to a more stable trans form. ^31^ Trans fatty acids negatively affect human health; for example, trans fatty acids increase LDL cholesterol and lower HDL cholesterol, thereby increasing the ratio of total cholesterol to HDL. In addition, trans fatty acids can increase blood triglycerides and reduce LDL particle size, which contributes to coronary heart disease. ^31^^-^^33^

In the present study, the resulting fish oil was used to improve the quality of fish feed. However, the influence of the significant elaidic acid content in fish oil on the fatty acid composition of fish feeds and the whole-body carcass, including the lipid profile in the serum of fish, remains complex and difficult to fully understand. Therefore, it is important to study the use of fish oil derived from the mesenteric tissue of Pangasius catfish processing waste to enrich Asian redtail catfish fry feed.

In this research, the levels of arachidonic acid (ARA, 20:4n-6) were found to be within 0.31% to 0.52%. The levels of docosahexaenoic acid (DHA, 22:6n-3) varied between 0.11% and 0.16%, while eicosapentaenoic acid (EPA, 20:5n-3) ranged from 0.10% to 0.15%. Despite being present in relatively low concentrations, ARA plays a significant role as a precursor in the synthesis of prostaglandins and is involved in regulating sex hormones. ^34^ Additionally, EPA and DHA also hold importance in promoting optimal fish growth and reproduction. ^35^^–^^38^ Utilizing offal from freshwater fish aquaculture as a supplement in fish diets could be a feasible alternative. ^39^^,^^40^

Moreover, the group size of fish exhibits lower levels of omega-3 fatty acids compared to omega-6 and omega-9 fatty acids (as indicated in Table 2). These groups of fatty acids play distinct roles: essential omega-3 fatty acids are primarily responsible for triggering anti-inflammatory responses, whereas omega-6 fatty acids tend to elicit pro-inflammatory reactions. On the other hand, non-essential omega-9 fatty acids play a crucial role as components in various metabolic pathways that could impact the risk of diseases. ^41^ The consequences of enriching fish feed with fish oil from Pangasius catfish waste on fingerlings' survival, growth, and the fatty acid composition in fish meat remain unclear. However, these intricate factors present us with challenges to enhance the future survival and growth of fish fry, including those of the Asian redtail catfish fry.

### Impact of fluctuating heating temperatures on the fatty acid composition of fish oil

Fish oil, a fatty component found in fish body tissue, is extracted through various methods. One of these methods is the dry rendering method, which involves subjecting the fish oil to a temperature treatment without the addition of water. ^42^^,^^43^

The distribution of fatty acids in fish oil subjected to varying temperatures is presented in Table 3. This finding aligns with previously reported data on milkfish ( *Chanos-chanos*) and catfish ( *Clarias* sp.), indicating higher levels of palmitic acid (C16:0, categorized as saturated fatty acids - SFA), oleate (18:1n-9, categorized as mono-unsaturated fatty acids - MUFA), and linoleic acid (C18:2n-6, categorized as poly-unsaturated fatty acids - PUFA) in both species. ^44^^,^^45^ Notably, substantial fluctuations in fatty acid composition were observed across the four temperature treatments, demonstrating statistically significant distinctions ( *p* < 0.05) among the temperature conditions.

**Table 3.** Fatty acid composition of Pangasius catfish oil with different heating temperatures.

Fatty acid (% ww/ww)	Heating temperatures			
	A (45 ^o^C)	B (60 ^o^C)	C (75 ^o^C)	D (90 ^o^C)
Undecanoic acid, C11:0	1.41 ± 0.01 ^a^	0.92 ± 0.01 ^b^	1.52 ± 0.02 ^c^	1.22 ± 0.00
Tridecanoic acid, C13:0	3.09 ± 0.01 ^a^	2.87 ± 0.02 ^b^	3.37 ± 0.02 ^c^	2.82 ± 0.01
Palmitic acid, C16:0	4.09 ± 0.02 ^a^	4.38 ± 0.03 ^b^	0.86 ± 0.02 ^c^	0.81 ± 0.01
Behenic acid, C22:0	1.40 ± 0.02 ^a^	1.22 ± 0.02 ^b^	1.42 ± 0.01 ^c^	1.31 ± 0.01
Heneicosanoic acid, C21:0	0.51 ± 0.03 ^a^	0.47 ± 0.02 ^b^	0.51 ± 0.01 ^c^	0.57 ± 0.02 ^d^
**Saturated fatty acids (SAFAs)**	**10.54 ± 0.11**	**9.87 ± 0.09**	**7.70 ± 0.07**	**6.73 ± 0.02**
Cis-10-Pentadecanoic acid, C15:1	8.56 ± 0.03 ^a^	8.73 ± 0.02 ^b^	8.77 ± 0.00 ^c^	8.13 ± 0.02 ^d^
Cis-10-Heptadecanoic acid, C17:1	3.15 ± 0.04 ^a^	3.03 ± 0.02 ^b^	3.32 ± 0.02 ^c^	3.07 ± 0.01 ^d^
Cis-11-Eicosenoic acid, C20:1	0.45 ± 0.00 ^a^	0.46 ± 0.01 ^b^	0.47 ± 0.01 ^c^	0.45 ± 0.01 ^ad^
Nervonic acid, C24:1	0.23 ± 0.00	0.24 ± 0.00	0.25 ± 0.01	0.25 ± 0.01
cis-9-Oleic acid (C18:1n9c)	36.24 ± 0.02	36.00 ± 0.04	34.88 ± 0.03	34.20 ± 0.05
Elaidic acid, C18:1n9t	14.18 ± 0.01 ^a^	15.18 ± 0.03 ^b^	15.62 ± 0.01 ^c^	16.34 ± 0.12 ^d^
**Mono-unsaturated fatty acids (MUFAs)**	**62.83 ± 0.09**	**63.65 ± 0.07**	**63.32 ± 0.05**	**62.46 ± 0.21**
Linolelaidic acid, C18:2n9t	8.68 ± 0.02 ^a^	7.53 ± 0.02 ^b^	8.50 ± 0.01 ^c^	7.95 ± 0.05
Linoleic acid, C18:2n6c	0.08 ± 0.00	0.08 ± 0.00	0.08 ± 0.00	0.09 ± 0.01
Arachidonic acid (ARA), 20:4n6	0.53 ± 0.00 ^a^	0.50 ± 0.01 ^b^	0.45 ± 0.02 ^c^	0.41 ± 0.01 ^d^
y-Linolenic acid, C18:3n6	0.85 ± 0.02 ^a^	0.83 ± 0.01 ^b^	0.84 ± 0.01 ^c^	0.83 ± 0.00 ^d^
cis-11, 14, 17-Eicosatrienoic acid Methyl Ester, (C20:3n3)	0.30 ± 0.00 ^a^	0.32 ± 0.00 ^b^	0.27 ± 0.01 ^c^	0.28 ± 0.00 ^d^
Eicosapentaenoic acid (20:5n3)	0.15 ± 0.00 ^a^	0.13 ± 0.00 ^b^	0.11 ± 0.01 ^c^	0.07 ± 0.00 ^c^
Docosahexaenoic acid (C22:6n3)	0.16 ± 0.01 ^a^	0.14 ± 0.00 ^b^	0.12 ± 0.00 ^c^	0.10 ± 0.01 ^d^
**Poly-unsaturated fatty acids (PUFAs)**	**10.67 ± 0.01**	**9.53 ± 0.02**	**9.30 ± 0.01**	**8.67 ± 0.01**
Unsaturated fatty acid	55.55	54.26	54.02	52.06
n-3 fatty acid	1.46	1.42	1.34	1.28
n-6 fatty acid	9.29	8.11	9.03	8.45
n-9 fatty acid	36.92	36.7	35.6	34.9

Mean ± SE (n = 3) with different letters in the same row are significantly different.

In this study, the SFA content was dominated by palmitic acid, which ranged from 0.81% to 4.09%. The higher the heating temperature was, the lower the palmitic acid content (see Table 3). The process of heating fish oil can affect the composition of the fatty acids in the oil, including the content of palmitic acid. ^44^ Chemical reactions in fish oil can occur more quickly at certain temperatures. Excessive heating or heating at too high a temperature can cause changes in the composition of fatty acids, including palmitic acid.

Palmitic acid is among the two dominant fatty acids and is commonly found in fish bodies, with high levels in various types of fish. ^45^ However, the findings by Sattang et al. ^13^ showed that in the SFA group, myristic acid (C14:0) and stearic acid (C18:0) played a more dominant role. The heating method can also affect changes in the fatty acid content. Slower or better-controlled heating may reduce the risk of drastic changes in the fatty acid content. ^46^

The content of elaidic acid at 45°C was 14.18%, which tended to increase to 16.34% at 90°C. On the other hand, the oleic acid content tended to decrease from 36.24% at 45°C to 34.2% at 90°C. Elaidic acid and oleic acid are geometric isomers of oleic acid. ^28^ Cis oleic acid is a more prooxidative factor than trans elaidic acid based on consumption of headspace oxygen under riboflavin photosensitization, while trans elaidic acid acted as a prooxidant for lipid hydroperoxides. ^47^

When fish oil is heated, especially at high temperatures, unsaturated fatty acids can undergo oxidation and geometrical isomerization changes, including the conversion of oleic acid to elaidic acid (see Table 3). This oxidation can produce unwanted compounds, such as free radicals, aldehydes, and other compounds that can damage nutrition and produce an unpleasant aroma or taste. ^48^

In this study, Pangasius catfish oil contained 6.73% to10.4% saturated fatty acids (SFAs), 62.46% to 63.65% mono-unsaturated fatty acids (MUFAs), and 8.67% to10.67% poly-unsaturated fatty acids (PUFAs). The content of eicosapentaenoic acid (EPA) is 0.07% to 0.15% of total fatty acids (FAs), while docosahexaenoic acid (DHA) is 0.10% to 0.16% of total fatty acids. Likewise, the fatty acid content of fresh water fish oils, such as Pangasius hybrid catfish and carp fillets, is lower in EPA and DHA. ^13^^,^^49^ Freshwater fish, including Pangasius catfish, red tail catfish, and carp, contain fewer essential unsaturated fatty acids (DHA, EPA) but are richer in amino acids than marine fish. ^37^^,^^50^^,^^51^

In this study, the content of arachidonic acid (20:4n6) in fish oil tended to decrease with increasing heating temperature. In addition, the content of eicosapentaenoic acid (20:5n3) and docosahexaenoic acid (C22:6n3) also decreased (see Table 3). This factor may be related to the arachidonic acid content at each heating temperature of fish oil. According to Jhonson and Bradfort ^41^ the most well-known bioactive lipid mediators include arachidonic acid (AA, C20:4n6), eicosapentaenoic acid (EPA, C22:5n3), and docosahexaenoic acid (DHA, C20:6n3). These three compounds are produced from essential precursors, namely linoleate (LA, C18:3n6) and α-linolenic acid (ALA, C18:3n3).

Omega-9 fatty acids, also known as oleic acid, are believed to play an important role in the metabolism of essential fatty acids in this study. This lipid has a role in regulating inflammatory processes both pro and anti, with its ability to stimulate enzymes and produce cytokines and other acute phase molecules. ^52^ According to the findings in this report, reducing your intake of omega-6 fatty acids (such as linoleic acid) from fish oil can increase the availability of omega-3 fatty acids. This in turn can reduce the ratio of omega-6/omega-3 fatty acids in fish oil (please see Table 3). In addition, the lower the ratio of omega-6/omega-3 fatty acids will reduce the pro-inflammatory response, and consequently reduce the risk of disease. ^53^

## Conclusion

The utilization of processed catfish waste, particularly its mesenteric tissue collected from diverse fish sizes in this study region, has been identified as a valuable resource for freshwater fish oil. Diverse fatty acid contents were observed in all fish samples within the three groups subjected to varying heating temperatures. The optimal fatty acid composition was found in fish oil from fish Group B (weighing 440 g to 685 g), which was heated to 45°C. Despite the significance of fish oil from catfish waste and mesenteric tissue, data on the chemical composition of this material remains limited. Thus, the reported fatty acid content data for different fish size groups and heating temperatures serve as a foundation for future research, addressing the challenges caused by the demand for fish oil in fish feed production.

## Data Availability

Figshare: Changes in the fatty acid profile of fish oil derived from Pangasius catfish ( *Pangasianodon hypophthalmus*) processing waste due to variations in fish size and heating temperatures. https://doi.org/10.6084/m9.figshare.24037500. ^54^ This project contains the following underlying data:
‐
Table 1. Presents the raw biometry data for Group A of Pangasius catfish samples ‐
Table 2. Presents the raw biometry data for Group B of Pangasius catfish samples ‐
Table 3. Presents the raw biometry data group C of Pangasius catfish ‐
Table 4. Raw data for the fatty acid composition of three groups of Pangasius catfish ‐
Table 5. Raw data fatty acid composition of Pangasius catfish oil with different heating temperatures Data are available under the terms of the Creative Commons Attribution 4.0 International license (CC-BY 4.0).
