# Supplementary material for: Changes in the fatty acid profile of fish oil derived from Pangasius catfish (Pangasianodon hypophthalmus) processing waste due to variations in fish size and heating temperatures
Source: F1000Res. 2023 Nov 23;12:1255. Originally published 2023 Oct 2. [Version 2] doi: 10.12688/f1000research.141714.2 (PMC12368491; doi:10.12688/f1000research.141714.2)
Supplement: Peer review history

# Reviewer report: Reviewer response for version 2

**DOI:** 10.5256/f1000research.159213.r397649

**Published:** 2025-08-27

**Authors:** Heru Sasongko

**Affiliations:** 1 Universitas Sebelas Maret, Surakarta, Indonesia

The study appears to have been conducted with considerable care and attention to detail. However, there are several aspects of the manuscript that may benefit from clarification and refinement in order to strengthen its overall clarity and scientific rigor. I respectfully offer the following suggestions:

1. There seems to be a lack of clarity between the title and the stated objectives, particularly regarding the variables and measurement dimensions of the fish (e.g., volume/weight). This is especially noticeable in the abstract, where the sentence: *"This study aimed to analyse the relationship between fresh weight and mesenteric weight in each group of fish and determine how variations in the size of catfish (Pangasianodon hypophthalmus) and heating temperature affect fatty acid profiles,"* could be more precisely aligned with the study’s focus.
2. In the background section, the discussion and citations related to fish feed types appear misaligned with the core objective of the research. Since variables such as feed type, rearing environment, fish size and age, extraction method, solvent, and temperature may each independently affect fatty acid content, the narrative should be revised to better reflect and support the study’s primary aim.
3. It would be helpful to emphasize the significance or potential impact of the study’s findings in the introduction, to highlight the research’s novelty and urgency.
4. The extraction methodology is not described in sufficient detail. While temperature ranges are provided, the method of heating (e.g., boiling, oven-drying, or other techniques) should be clearly specified to ensure reproducibility and methodological clarity.
5. The justification for the observed variations in fatty acid profiles particularly omega-3, omega-6, and omega-9 in relation to fish size appears inconsistent. A more detailed discussion supported by relevant literature would enhance the interpretability of these findings.
6. The influence of heating temperature on fatty acid levels is briefly noted, particularly the decrease in omega-3 and omega-9 levels (with omega-6 remaining relatively unaffected). Further elaboration and comparison with existing studies would help to contextualize and validate these observations.

Is the work clearly and accurately presented and does it cite the current literature?

Partly

If applicable, is the statistical analysis and its interpretation appropriate?

Yes

Are all the source data underlying the results available to ensure full reproducibility?

Yes

Is the study design appropriate and is the work technically sound?

Yes

Are the conclusions drawn adequately supported by the results?

Partly

Are sufficient details of methods and analysis provided to allow replication by others?

Partly

Reviewer Expertise:

endocrine disorder, pharmacy, pharmacology

I confirm that I have read this submission and believe that I have an appropriate level of expertise to confirm that it is of an acceptable scientific standard, however I have significant reservations, as outlined above.

# Reviewer report: Reviewer response for version 2

**DOI:** 10.5256/f1000research.159213.r397650

**Published:** 2025-08-25

**Authors:** Cosmas Nathanailides

**Affiliations:** 1 University of Ioannina, Ioannina, Greece

The gonadosomatic index (GSI) and visceral content index are both reported in Table 1, with values given for small, medium, and large fish as percentages of body weight. However, while these indices are presented numerically, the sexual maturation stage of the fish is not discussed or interpreted in the context of the GSI values. There is also no mention of histological or macroscopic confirmation of gonadal development stages, making it difficult to determine whether the GSI differences reflect actual reproductive maturity. This weakens the ability to draw conclusions about physiological status, which—as you noted earlier—can significantly influence lipid metabolism and fatty acid composition

 Furthermore, the study does not report the age, sex, or sexual maturation stage of the fish in the different size groups. These are fundamental biological variables known to influence lipid metabolism and fatty acid deposition. Without controlling for or at least acknowledging these factors, it becomes unclear whether the observed differences in fatty acid composition are due to body size, physiological status, or other uncontrolled variables. The omission of this information significantly undermines the internal validity of the study and compromises the interpretability of its findings. These design and methodological shortcomings should be addressed in future work to ensure that conclusions are supported by robust and biologically meaningful data.

 Beyond the experimental design issues, the experimental design also presents significant limitations. The methodology of fatty acid analysis lacks clarity. The use of pre-extraction heating at 60°C likely altered the native fatty acid composition through oxidation and isomerization, particularly of unsaturated fatty acids. Furthermore, subjecting the extracted oil to additional heating before GC–MS analysis confounds biological differences with thermal degradation effects. The transmethylation procedure is insufficiently described, omitting key details such as reaction conditions and antioxidant use. Most notably, the GC–MS protocol is inadequately reported, lacking internal standards and instrumental specifications essential for accurate identification and quantification. These methodological gaps undermine the validity and reproducibility of the findings and should be addressed.

 It is unclear why the authors report the fatty acid composition of visceral fat in Table 5 but provide no data on the flesh tissue, which is the primary edible portion. This omission leaves a significant gap in understanding how body size and thermal processing affect the nutritional quality of the fish.

 The use of only a few samples for Pearson correlation analysis raises concerns about the reliability and validity of the results. A limited sample size reduces statistical power, increases the likelihood of random error, and can produce unstable or misleading correlation coefficients.

 The authors should clarify what proportion of the visceral tissue is represented by mesenteric fat. Providing this information will enable to asses the relation to the overall visceral yield and improve the understanding of its nutritional and economic significance.

Is the work clearly and accurately presented and does it cite the current literature?

No

If applicable, is the statistical analysis and its interpretation appropriate?

Partly

Are all the source data underlying the results available to ensure full reproducibility?

Partly

Is the study design appropriate and is the work technically sound?

No

Are the conclusions drawn adequately supported by the results?

Partly

Are sufficient details of methods and analysis provided to allow replication by others?

No

Reviewer Expertise:

Fish physiology

I confirm that I have read this submission and believe that I have an appropriate level of expertise to state that I do not consider it to be of an acceptable scientific standard, for reasons outlined above.

# Reviewer report: Reviewer response for version 2

**DOI:** 10.5256/f1000research.159213.r397653

**Published:** 2025-08-20

**Authors:** Suprakash Chakma

**Affiliations:** 1 Patuakhali Science and Technology University, Patuakhali, Bangladesh

Reviewer Comments: Minor revision.

 ‘Changes in the fatty acid profile of fish oil derived from Pangasius catfish ( *Pangasianodon hypophthalmus*) processing waste due to variations in fish size and heating temperatures’

 The present work is interesting in present and future perspective. However, after correction of the following comments, the manuscript can be accepted for the publication.

 Reviewer Comments:****

 There are some points to note

 Please adhere to the publication guidelines:

 Introduction:

 In general, the information is well presented, and the ideas are clear, giving an adequate introduction to the work.

 Abstract:

 I recommend and checked the abstract to ensure that it provides a clear overview of the impact of the study. Furthermore, conclusion should give more emphasize to rewrite again in future perspective.

 Materials and Methods:

 As the specimen is animal. Therefore, the authors must provide ethical statement from the ethical institutions.

1. Prior to the measurement of morphological properties, authors did anesthesia the species?
2. Must add total procedure of fatty acids profile analysis to clear to the readers.

Statistical data analysis

1. It is well indicated that all variables were given as mean±SD. It is very clearly defined that the data analysis was in good analysis and adjusted with the present study.

Results

 This section is well described. Carefully review to correct spelling and writing details.

 Discussion:

 Although the results shown are well written and interesting, I think it is necessary to go deeper in the discussion since the arguments are based on other species, without comparing with data obtained from relevant species, in addition to the fact that it is necessary to carry out a more in-depth discussion to increase the impact and the beneficial effects of the results obtained. You can cite the following paper in case of the deeply discussion:

1. Nutritional Profiling of Wild (Pangasius pangasius) and Farmed (Pangasius hypophthalmus) Pangasius Catfish with Implications to Human Health

Conclusion:

 It is well presented but in a very general way. Be careful to base conclusions on results obtained without speculation.

 References:

 All references have been justified. Although, authors must follow journal rules and guidelines. But ensured to add the following citation:

1. Nutritional Profiling of Wild (Pangasius pangasius) and Farmed (Pangasius hypophthalmus) Pangasius Catfish with Implications to Human Health

Is the work clearly and accurately presented and does it cite the current literature?

Yes

If applicable, is the statistical analysis and its interpretation appropriate?

Yes

Are all the source data underlying the results available to ensure full reproducibility?

Partly

Is the study design appropriate and is the work technically sound?

Yes

Are the conclusions drawn adequately supported by the results?

Partly

Are sufficient details of methods and analysis provided to allow replication by others?

Partly

Reviewer Expertise:

Food Processing and Technology, Fish oil and marine bioacitve compounds, Food Science and Food Microbiology

I confirm that I have read this submission and believe that I have an appropriate level of expertise to confirm that it is of an acceptable scientific standard.

## References

[1] : Nutritional Profiling of Wild (Pangasius pangasius) and Farmed (Pangasius hypophthalmus) Pangasius Catfish with Implications to Human Health. *Fishes* .2022;7(6) : 10.3390/fishes7060309 10.3390/fishes7060309

# Reviewer report: Reviewer response for version 2

**DOI:** 10.5256/f1000research.159213.r226600

**Published:** 2024-01-29

**Authors:** Pande Gde Sasmita Julyantoro

**Affiliations:** 1 Faculty of Marine Science and Fisheries, Universitas Udayana, Bali Province, Indonesia

This manuscript has been prepared well and systematically. The references are also up to date. 

 Currently, research on the sources of fish oil as a component of making fish feed has become important because of the limited sources of oil that come directly from the catch. This will be very relevant to fill the lack of fish oil sources to meet the increasing demand for fish feed in the aquaculture industry.

 This study examines waste from catfish processing, especially the mesenteric tissue which can be used as a source of fish oil with different sizes and heating temperatures and their influence on the fatty acid profile.

 In general, this manuscript has been prepared well and systematically. The references are also up to date. The minor point that needs to be revised is the clear explanation of the fatty acid composition. It would be better to mention explicitly why the most optimum fatty acid composition is obtained from fish group B. It is very interesting to discuss scientifically because there may be something related to the fatty acid composition in terms of the development of the mesenteric tissue since the optimum fatty acid composition is not obtained from Group C, which is heavier than Group B.

Is the work clearly and accurately presented and does it cite the current literature?

Yes

If applicable, is the statistical analysis and its interpretation appropriate?

Yes

Are all the source data underlying the results available to ensure full reproducibility?

Yes

Is the study design appropriate and is the work technically sound?

Yes

Are the conclusions drawn adequately supported by the results?

Yes

Are sufficient details of methods and analysis provided to allow replication by others?

Yes

Reviewer Expertise:

Aquaculture, Aquatic microbiology

I confirm that I have read this submission and believe that I have an appropriate level of expertise to confirm that it is of an acceptable scientific standard.

# Response

**Published:** 2024-02-12

**Authors:** Netti Aryani

**Affiliations:** Universitas Riau, Indonesia

The author will include an underlined sentence in this revision to strengthen the statement that group B fish have a higher fatty acid content.

 In the different size categories of fish, significant quantities of mono-unsaturated fatty acids (MUFAs) were present, particularly oleic acid. The respective proportions of oleic acid were 32.72% for Group A, 36.61% for Group B, and  34.55% for Group C. The high levels of MUFA and PUFA in the mesenteric tissue of group B fish may be caused by the higher condition factor value (2.1 ± 0.28) compared to groups A (1.58 ± 0.02) and C (1.98 ± 0.04 ). The higher condition factor value indicates an adequate level of feeding and appropriate environmental conditions, as well as providing added value to MUFAs and PUFAs, especially oleic acid ^21^. On the other hand, providing quality food can influence the content of MUFAs and PUFAs in Nile tilapia ( *Oreochromis niloticus*) fillets ^25^ . Additionally, elaidic acid concentrations ranged from 13.72% to 16.31%, surpassing other classified MUFA types (refer to Table 2). This discovery aligns with previous data from *Sardinella lemuru* and *Cyprinus carp*io fish oil research, ^24,27^ in which oil from both species exhibited comparatively elevated levels of oleic and elaidic acid. Unlike the results found in research by Sattang et al., ^13^. fish oil derived from the adipose tissue of a freshwater hybrid catfish ( *P. gigas *x *P. hypophthalmus*) has an oleic acid content of 42.27%, while the elaidic acid content is low, namely, 0.25%.
